# Clinical and genetic analyses of a Dutch cohort of 40 patients with a nephronophthisis-related ciliopathy

**DOI:** 10.1007/s00467-018-3958-7

**Published:** 2018-07-05

**Authors:** Marijn F. Stokman, Bert van der Zwaag, Nicole C. A. J. van de Kar, Mieke M. van Haelst, Albertien M. van Eerde, Joost W. van der Heijden, Hester Y. Kroes, Elly Ippel, Annelien J. A. Schulp, Koen L. van Gassen, Iris A. L. M. van Rooij, Rachel H. Giles, Philip L. Beales, Ronald Roepman, Heleen H. Arts, Ernie M. H. F. Bongers, Kirsten Y. Renkema, Nine V. A. M. Knoers, Jeroen van Reeuwijk, Marc R. Lilien

**Affiliations:** 1 Department of Genetics, Center for Molecular Medicine, University Medical Center Utrecht, Utrecht University, Utrecht, The Netherlands; 2 Department of Pediatric Nephrology, Radboud University Medical Center, Nijmegen, The Netherlands; 3 Department of Clinical Genetics, VU University Medical Center, Amsterdam, The Netherlands; 4 Department of Clinical Genetics, Academic Medical Center, Amsterdam, The Netherlands; 5 Department of Nephrology, VU University Medical Center, Amsterdam, The Netherlands; 6 Department of Pediatric Nephrology, Wilhelmina Children’s Hospital, University Medical Center Utrecht, Utrecht, The Netherlands; 7 Radboud Institute for Health Sciences, Department for Health Evidence, Radboud University Medical Center, Nijmegen, The Netherlands; 8 Department of Nephrology and Hypertension, University Medical Center Utrecht, Regenerative Medicine Center, University of Utrecht, Utrecht, The Netherlands; 9 Genetics and Genomic Medicine, UCL Great Ormond Street Institute of Child Health, London, UK; 10 Department of Human Genetics, Radboud University Medical Center, Nijmegen, The Netherlands; 11 Radboud Institute for Molecular Life Sciences, Radboud University Medical Center, Nijmegen, The Netherlands; 12 Department of Pathology and Molecular Medicine, Hamilton Health Sciences, McMaster University, West Hamilton, Canada

**Keywords:** Nephronophthisis, Pediatric kidney disease, Ciliopathy, Clinical registry, Gene-phenotype association

## Abstract

**Background:**

Nephronophthisis is an autosomal recessive ciliopathy and important cause of end-stage renal disease (ESRD) in children and young adults. Diagnostic delay is frequent. This study investigates clinical characteristics, initial symptoms, and genetic defects in a cohort with nephronophthisis-related ciliopathy, to improve early detection and genetic counseling.

**Methods:**

Forty patients from 36 families with nephronophthisis-related ciliopathy were recruited at university medical centers and online. Comprehensive clinical and genotypic data were recorded. Patients without molecular diagnosis were offered genetic analysis.

**Results:**

Of 40 patients, 45% had isolated nephronophthisis, 48% syndromic diagnosis, and 7% nephronophthisis with extrarenal features not constituting a recognizable syndrome. Patients developed ESRD at median 13 years (range 5–47). Median age of symptom onset was 9 years in both isolated and syndromic forms (range 5–26 vs. 5–33). Common presenting symptoms were fatigue (42%), polydipsia/polyuria (33%), and hypertension (21%). Renal ultrasound showed small-to-normal-sized kidneys, increased echogenicity (65%), cysts (43%), and abnormal corticomedullary differentiation (32%). Renal biopsies in eight patients showed nonspecific signs of chronic kidney disease (CKD). Twenty-three patients (58%) had genetic diagnosis upon inclusion. Thirteen of those without a genetic diagnosis gave consent for genetic testing, and a cause was identified in five (38%).

**Conclusions:**

Nephronophthisis is genetically and phenotypically heterogeneous and should be considered in children and young adults presenting with persistent fatigue and polyuria, and in all patients with unexplained CKD. As symptom onset can occur into adulthood, presymptomatic monitoring of kidney function in syndromic ciliopathy patients should continue until at least age 30.

**Electronic supplementary material:**

The online version of this article (10.1007/s00467-018-3958-7) contains supplementary material, which is available to authorized users.

## Introduction

Nephronophthisis (NPH) is an autosomal recessive kidney disease that is responsible for up to 15% of pediatric patients with end-stage renal disease (ESRD) [1–3]. In The Netherlands, this corresponds to an estimated one to five children with ESRD per year [4]. NPH is characterized by a renal concentration defect, chronic tubulointerstitial nephritis, renal cysts in 50% of patients, and progression to ESRD before age 30 years [5–7]. Three clinical subtypes are recognized based on age of onset. The most prevalent form, juvenile NPH, results in ESRD at a median age of 13 years [3, 7–9]. NPH can occur in an isolated form and, in 10 to 15% of cases, as part of a multisystem disorder, for example Senior-Løken syndrome and Joubert syndrome. Both isolated and syndromic NPH are caused by mutations in genes encoding proteins that localize to primary cilia. These mutations result in structural and functional ciliary defects, and disruption of essential ciliary and nuclear signaling pathways. Hence, NPH is considered a ciliopathy.

The diagnosis of NPH is based on clinical findings, renal ultrasound findings, and a family history compatible with autosomal recessive inheritance. The average diagnostic delay in isolated NPH is 3.5 years because of nonspecific presenting symptoms including polydipsia and polyuria, secondary enuresis, growth retardation, and anemia refractory to iron therapy [7]. In addition, causal mutations in one of the 20 NPH-associated genes are detected in less than 50% of patients [10–12]. Although recent progress has been made in characterizing the clinical spectrum of NPH [6, 7, 13, 14], the absence of solid genotype-phenotype correlations currently limits prognostic counseling of NPH patients. Therefore, analysis of presenting symptoms, renal and extrarenal phenotypes, and associated genetic defects in a well-defined population of NPH patients is warranted.

Here we report clinical characteristics and genetic findings in a cohort of 40 Dutch patients from 36 families with a nephronophthisis-related ciliopathy (NPH-RC). We assess progression of chronic kidney disease (CKD) in isolated and syndromic forms of NPH and discuss the initial signs and symptoms of kidney disease that could point towards NPH in an early disease stage. Renal and extrarenal phenotypes and associated genetic defects are analyzed. Finally, our results suggest the limited diagnostic value of a renal biopsy in advanced stages of CKD and point towards the utility of early molecular testing in diagnostics of NPH.

## Methods

### Patients

Patients were recruited at all eight university medical centers in The Netherlands, via the Dutch Kidney Patient Association and the Dutch Joubert Syndrome Patient and Parents’ Network, and via the webpage www.kouncil.nl. Patients were included in the AGORA (Aetiologic research into Genetic and Occupational/environmental Risk factors for Anomalies in children) data- and biobank from March 2014 until May 2017 at the Department of Genetics and the Department of Pediatric Nephrology of the University Medical Center Utrecht, the Department of Human Genetics of the Radboudumc Nijmegen, and the Department of Nephrology of the VU University Medical Center Amsterdam. Approval by the regional Committee on Research involving human subjects (CMO Arnhem-Nijmegen 2006/048) was obtained. Informed consent was available for all subjects for access to medical records, DNA testing, and/or collection of biomaterial including urine, fibroblasts, and/or deciduous teeth.

Inclusion criteria were a molecularly confirmed diagnosis of NPH-RC or suspected NPH-RC. NPH-RC was defined as isolated NPH, NPH with extrarenal features that do not constitute a recognizable syndrome, and syndromic NPH. Suspected NPH-RC was based on the presence of CKD stages 2–5 in all patients [15], a family history compatible with autosomal recessive inheritance, and additional extrarenal features associated with a ciliopathy and/or clinical characteristics of NPH including signs of a renal concentration defect (e.g., polyuria), renal ultrasound showing parenchymal damage (e.g., increased echogenicity) but absence of congenital anomalies of the kidneys and urinary tract that could explain the phenotype, urinalysis showing absence of hematuria, erythrocyte casts and proteinuria in the nephrotic range, absence of recurrent urinary tract infections, and exclusion of other causes of early-onset renal failure. Cases in which the clinical diagnosis was ambiguous were discussed with a pediatric nephrologist (M. L.) and a clinical geneticist (N. K.) until consensus was reached. We included five patients with Bardet-Biedl syndrome and a nephronophthisis phenotype; however, the classification of Bardet-Biedl syndrome as NPH-RC is not consistent across literature [6, 11]. Siblings of patients with molecularly confirmed NPH-RC who had the same biallelic mutations in an NPH-RC-associated gene and who showed some but not all clinical features were included for the analysis of initial phenotypic features of NPH. In addition to 40 patients with NPH-RC, we included 12 patients with a ciliopathy and renal features that did not fulfill clinical criteria of NPH to assess the phenotypic spectrum of renal ciliopathies.

### The Nephronophthisis Registry

To record detailed clinical and genetic data from patients with NPH-RC, we developed a multicenter Nephronophthisis Registry using Castor EDC (Castor Electronic Data Capture, Ciwit BV, Amsterdam, The Netherlands). Access to electronic medical records or paper copies of medical records was requested for all patients. For standardized data collection, a set of clinical parameters and definitions was established by a team of clinical experts from relevant fields including pediatric nephrology, clinical genetics, and clinical molecular genetics. Consensus was reached on all parameters. Human Phenotype Ontology (HPO) terms were used for most of the clinical terms recorded to phenotype the patients, thereby also providing a basis for future integration with clinical data from other nephronophthisis registries and for clinical matching of patients in HPO-centered registries of rare diseases [16]. Case report forms were structured in categories including medical history, physical examination, ophthalmological examination, and genetic analysis. For longitudinal data, we developed report forms for laboratory results, medication, physical re-examination, histological examination, and imaging. Syndrome diagnoses were based on clinical data on renal and extrarenal phenotypes (e.g., presence of molar tooth sign on brain imaging in Joubert syndrome) according to internationally recognized diagnostic criteria [17–20], in combination with the assessment of the referring physician.

### Genetic analyses

Of 17 patients without a molecular diagnosis upon inclusion, 13 patients from 12 families gave consent for genetic testing. Targeted deletion/duplication analysis was performed to assess the occurrence of *NPHP1* deletions in patients with isolated NPH, Senior-Løken syndrome, and Joubert syndrome, and to assess copy number variations in ciliary genes in which we identified single heterozygous point mutations (Supplementary Methods S1). Targeted next-generation sequencing using a gene panel containing 15 genes associated with NPH was performed in one patient (10:44). Whole-exome sequencing (Supplementary Methods S2) was subsequently performed in this patient and 10 additional patients with no deletion or a heterozygous deletion in *NPHP1*, including a patient with Bardet-Biedl-like syndrome in whom biallelic *NPHP1* deletions were unlikely to be causal.

### Statistical analyses

Clinical and genetic data from the Nephronophthisis Registry were exported to Excel and analyzed using GraphPad Prism (version 7.02).

## Results

### Phenotypic variability in renal ciliopathies

**Fig. 1:**
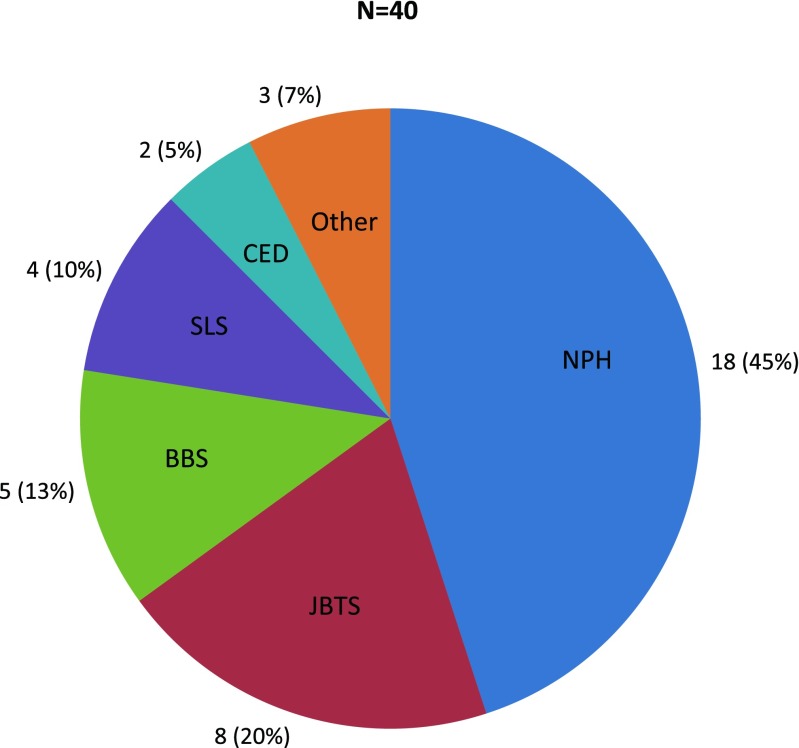
Relative frequencies of (nephronophthisis-related ciliopathy) NPH-RC phenotypes in the cohort. *NPH* isolated nephronophthisis, *JBTS* Joubert syndrome, *BBS* Bardet-Biedl syndrome, *SLS* Senior-Løken syndrome, *CED* cranioectodermal dysplasia

Forty patients from 36 families with NPH-RC were recruited, including patients with isolated NPH (*n* = 18), Joubert syndrome (*n* = 8), Bardet-Biedl syndrome (*n* = 5), Senior-Løken syndrome (*n* = 4), cranioectodermal dysplasia (*n* = 2), and NPH-RC phenotypes that did not constitute a recognizable syndrome (*n* = 3) (Fig. 1). Twenty-two out of 40 patients (55%) had extrarenal ciliopathy-associated features including impaired vision (16/40; 40%), oculomotor abnormalities (10/33; 30%), and developmental delay (13/36; 36%), indicating enrichment of syndromic forms of NPH-RC in our cohort (Supplementary Table S1). Table 1 depicts the frequency of occurrence of clinical features per causative gene for 40 patients.

**Table 1:** Genetic and phenotypic findings in 40 patients with NPH-RC

Gene	Patients	Families	Median age (range)^a^ (years)	Visual impairment^b^	Oculomotor abnormalities	Developmental delay	Liver abnormalities	Congenital heart defect	Narrow thorax	Polydactyly	Obesity
*NPHP1*	*n* = 16	*n* = 14	21 (9–62)	19% (3/16)	7% (1/14)	7% (1/14)	0% (0/8)	8% (1/13)	0% (0/14)	0% (0/14)	13% (2/16)
*NPHP4*	*n* = 3	*n* = 3	19 (11–41)	0% (0/3)	0% (0/3)	0% (0/3)	0% (0/1)	0% (0/2)	0% (0/1)	0% (0/2)	0% (0/3)
*WDR35*	*n* = 3	*n* = 2	17 (12–33)	33% (1/3)	33% (1/3)	0% (0/3)	50% (1/2)	0% (0/3)	67% (2/3)	33% (1/3)	33% (1/3)
*BBS1*	*n* = 2	*n* = 1	18 (16–20)	100% (2/2)	0% (0/1)	100% (2/2)	0% (0/0)	0% (0/0)	0% (0/2)	0% (0/2)	100% (2/2)
*AHI1*	*n* = 1	*n* = 1	25	100% (1/1)	100% (1/1)	100% (1/1)	0% (0/1)	0% (0/1)	0% (0/1)	0% (0/1)	0% (0/1)
*BBS10*	*n* = 1	*n* = 1	13	100% (1/1)	NA	100% (1/1)	0% (0/1)	0% (0/1)	0% (0/1)	100% (1/1)	100% (1/1)
*IQCB1*	*n* = 1	*n* = 1	29	100% (1/1)	100% (1/1)	0% (0/1)	NA	NA	0% (0/1)	0% (0/1)	0% (0/1)
*OFD1*	*n* = 1	*n* = 1	12	0% (0/1)	100% (1/1)	100% (1/1)	NA	0% (0/1)	0% (0/1)	0% (0/1)	0% (0/1)
Unknown^c^	*n* = 12	*n* = 12	21 (15–54)	58% (7/12)	56% (5/9)	70% (7/10)	44% (4/9)	20% (1/5)	0% (0/6)	29% (2/7)	33% (3/9)
Total	*n* = 40	*n* = 36	19 (9–62)	40% (16/40)	30% (10/33)	36% (13/36)	23% (5/22)	8% (2/26)	7% (2/30)	13% (4/32)	24% (9/37)

*NA* not available; *NPH-RC* nephronophthisis-related ciliopathy

^a^When range is not reported, the value is based on one patient

^b^Visual symptoms include night blindness, retinitis pigmentosa, constricted visual fields, and ocular coloboma

^c^Genetic cause not identified

Patients developed ESRD at a median age of 13 years (range 5–47 years). The median age of symptom onset was 9 years in both isolated and syndromic forms of NPH (range 5–26 vs. 5–33 years respectively). Median time between reported symptom onset and ESRD was 1 year (range 0–8 years) in patients with isolated NPH (*n* = 11) and 5.5 years (range 0–14 years) in patients with syndromic forms of NPH (*n* = 4). Four out of 18 patients with isolated NPH (22%) did not yet have ESRD at the time of inclusion (median age 21 years; range 17–41 years), although two patients had at least CKD stage 3. In contrast, 10/20 patients with syndromic forms of NPH (50%) did not have ESRD at the time of inclusion (median age 15.5 years; range 10–25 years) and three patients had at least CKD stage 3. More longitudinal data will be collected to determine the rate of CKD progression in patients with isolated and syndromic forms of NPH.

To evaluate the range of renal ciliopathy-associated phenotypes, we included 12 patients with a renal ciliopathy without sufficient evidence for NPH. Phenotypes included perinatally lethal renal cystic hypodysplasia and renal insufficiency with proteinuria (Supplementary Results S1 and Supplementary Table S2).

### Fatigue and polyuria are presenting symptoms of NPH

**Fig. 2:**
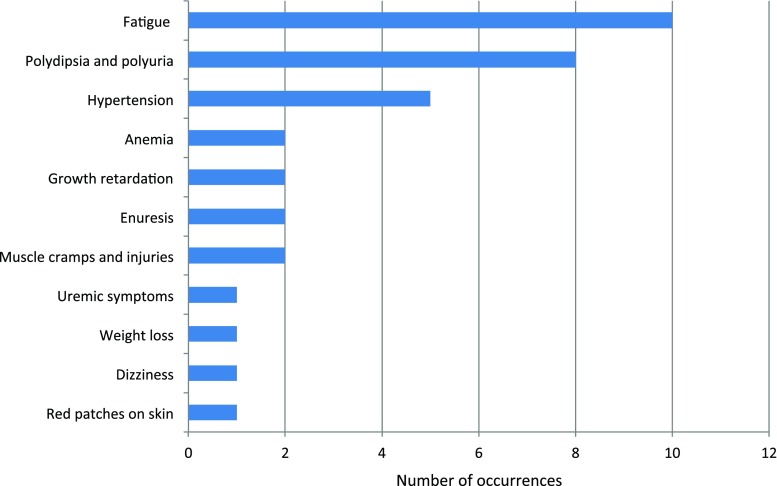
Presenting signs and symptoms of nephronophthisis. Presenting signs and symptoms of nephronophthisis (NPH) by frequency of occurrence. Data was available for 24 patients. For ten patients, more than one presenting sign or symptom was reported

To assess presenting symptoms of NPH, we reviewed medical records and conducted patient interviews. Presenting symptoms were documented for 24/40 patients. The most frequently reported initial signs and symptoms were fatigue (10/24; 42%), polydipsia and polyuria (8/24; 33%) and hypertension (5/24; 21%) (Fig. 2 and Supplementary Table S3). Anemia (2/24; 8%), growth retardation (2/24; 8%), enuresis (2/24; 8%), and uremic symptoms (1/24; 4%) occurred less frequently. Novel reported presenting symptoms included muscle cramps and injuries (*n* = 2), dizziness (*n* = 1), red patches on the skin (*n* = 1), and weight loss (*n* = 1). In addition, a patient with Joubert syndrome caused by *NPHP1* mutations (34:73) reported muscle cramps after NPH was diagnosed. Based on these findings, persistent fatigue and muscle complaints in children should prompt evaluation of kidney function in addition to the classic presenting symptoms of NPH.

### Increased echogenicity is a more prevalent finding than renal cysts in NPH-RC

Overall occurring symptoms were analyzed in 40 patients with NPH-RC. The most frequently reported symptoms were polydipsia and polyuria (18/25; 72% and 15/25; 60%, respectively) and enuresis (4/19; 21%) (Table 2 and Supplementary Table S3). Fifteen out of 23 patients (65%) had normal-sized kidneys on renal ultrasound and eight patients (35%) had at least one small kidney (size < 5th percentile) (reference values: [21, 22]). Small- to normal-sized kidneys is a typical finding in NPH [6, 7, 23]. Additional ultrasound findings included increased echogenicity (17/26; 65%), renal cysts (13/30; 43%), and abnormal corticomedullary differentiation (7/22; 32%). Increased echogenicity was the most prevalent finding on renal ultrasound and could therefore be considered a more sensitive finding for nephronophthisis than renal cysts.

**Table 2:** Associations between gene and renal phenotype

Gene	Patients	Families	Median age (range)^a^ (years)	Median age symptom onset (range)^a^(years)	Median age ESRD (range)^a^(years)	Median time to ESRD (range)^a,b^(years)	Polyuria	Polydipsia	Enuresis	Renal ultrasound findings		
										Cysts	Abnormal corticomedullary differentiation	Increased echogenicity
*NPHP1*	*n* = 16	*n* = 14	21 (9–62)	9 (6–26)	14 (7–28)	1 (0–8)	90% (9/10)	80% (8/10)	38% (3/8)	33% (4/12)	38% (3/8)	70% (7/10)
*NPHP4*	*n* = 3	*n* = 3	19 (11–41)	16 (8–24)	8	0	33% (1/3)	67% (2/3)	0% (0/3)	50% (1/2)	50% (1/2)	100% (2/2)
*WDR35*	*n* = 3	*n* = 2	17 (12–33)	9	12	3	67% (2/3)	50% (1/2)	0% (0/2)	67% (2/3)	33% (1/3)	100% (2/2)
*BBS1*	*n* = 2	*n* = 1	18 (16–20)	NA	–	NA	0% (0/1)	0% (0/1)	0% (0/1)	50% (1/2)	0% (0/2)	0% (0/2)
*AHI1*	*n* = 1	*n* = 1	25	14	–	NA	0% (0/1)	0% (0/1)	0% (0/1)	0% (0/1)	0% (0/1)	0% (0/1)
*BBS10*	*n* = 1	*n* = 1	13	NA	–	NA	100% (1/1)	100% (1/1)	0% (0/1)	100% (1/1)	100% (1/1)	100% (1/1)
*IQCB1*	*n* = 1	*n* = 1	29	NA	10	NA	NA	NA	NA	NA	NA	NA
*OFD1*	*n* = 1	*n* = 1	12	5	5	0	NA	NA	NA	0% (0/1)	0% (0/1)	100% (1/1)
Unknown	*n* = 12	*n* = 12	21 (15–54)	13 (5–33)	14 (8–47)	11 (8–14)	33% (2/6)	86% (6/7)	33% (1/3)	50% (4/8)	17% (1/6)	57% (4/7)
Total	*n* = 40	*n* = 36	19 (9–62)	9 (5–33)	13 (5–47)	1 (0–14)	60% (15/25)	72% (18/25)	21% (4/19)	43% (13/30)	32% (7/22)	65% (17/26)

*ESRD* end-stage renal disease; *−* absent, no ESRD; *NA* not available

^a^When range is not reported, the value is based on one patient

^b^Patient age at CKD stage 5 was used as the age at ESRD. If this was not available, the youngest reported age at the start of dialysis/transplantation was used. Median time to ESRD could be an underestimate because patients who did not develop ESRD were not included in this calculation

### Histological findings are frequently nonspecific in NPH

Renal biopsy was performed in 8/40 patients. Insufficient material was obtained from two patients, resulting in a second biopsy for one patient. Tubulointerstitial nephritis was a finding in 4/7 biopsies and tubular atrophy in 3/7. Renal cysts were reported in none of the patients. Instead, glomerulosclerosis was seen in 5/7 biopsies. Biopsies were taken in advanced stages of NPH (CKD stage ≥ 3 for all patients except patient 11:45), when differentiating findings such as thickening of tubular basement membranes and cysts on the corticomedullary border were expected to be no longer detectable. These findings suggest that the diagnostic value of a renal biopsy is limited in advanced stages of CKD, which is often already present when the diagnosis of NPH is first suspected.

### *NPHP1* mutations in 38% of tested patients

**Fig. 3:**
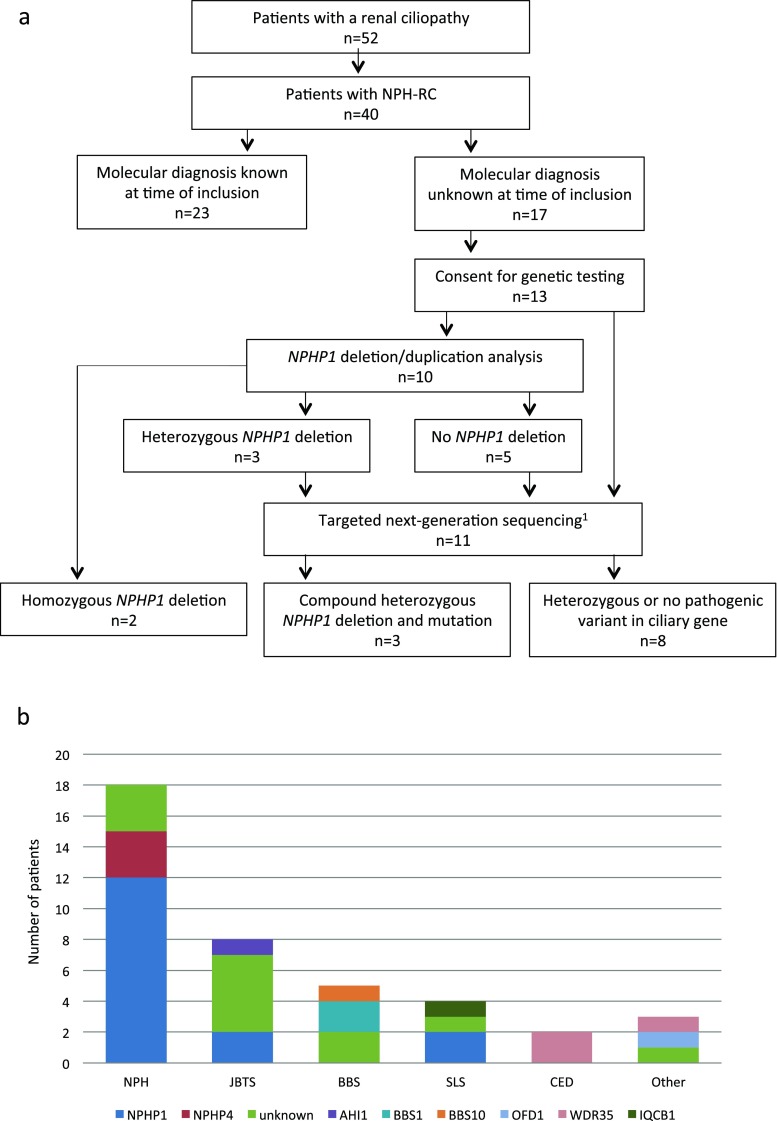
Clinical and genetic characterization of the cohort. **a** Genetic diagnostic testing strategy. (1) Targeted next-generation sequencing included a multi-gene panel and/or whole-exome sequencing. **b** Prevalence of causal genes per nephronophthisis-related ciliopathy (NPH-RC) phenotype. *NPH* isolated nephronophthisis, *JBTS* Joubert syndrome, *BBS* Bardet-Biedl syndrome, *SLS* Senior-Løken syndrome, *CED* cranioectodermal dysplasia

Twenty-three out of 40 patients (58%) had a (likely) molecular diagnosis at the time of inclusion (Supplementary Table S4). We performed genetic testing for 13 patients from 12 families without a molecular diagnosis, including genotyping, bioinformatics analysis, and/or functional assays for the interpretation of genetic variants (Fig. 3a). A biallelic mutation in *NPHP1* was detected in five out of 13 patients (38%), of whom two patients had a homozygous deletion and three patients had a compound heterozygous deletion and mutation (Table 3). Consequently, *NPHP1* was the most frequently mutated gene in our cohort (16/36 patients (44%); Fig. 3b), which is consistent with literature [10].

**Table 3:** Genetic findings in 13 NPH-RC patients

ID	Gene	Zygosity	Accession no.	c.Position	p.Position	HGMD accession	Polyphen-2	Mut Tast	SIFT	Gerp	ExAC^a^	Clinical diagnosis
6:39	*NPHP1*	Hom	NM_000272.3	c.1-?_2034+?del	p.(?)	NA	NA	NA	NA	NA	NA	NPH
24:60	*NPHP1*	Hom	NM_000272.3	c.1-?_2034+?del	p.(?)	NA	NA	NA	NA	NA	NA	NPH
9:42 9:43	*NPHP1*	Comp het	NM_000272.3	c.1-?_2034+?del;	p.(?)	NA	NA	NA	NA	NA	NA	NPH
				c.1039C>T	p.Arg347*	CM080456	NA	DC	NA	NA	NA	
11:45	*NPHP1*	Comp het	NM_000272.3	Deletion;	p.(?);	NA	NA	NA	NA	NA	NA	SLS
				c.1027G>A	p.Gly343Arg	CM066932	Probably damaging	DC	NA	5.79	13/0/119452	
23:58	*TMEM67*	Comp het	NM_153704.5	c.870-5del^b^;	p.(?)	NA	NA	NA	NA	NA	NA	Other (BBS-like)
				c.1379G>C	p.Arg460Thr	NA	Benign	DC	NA	NA	36/0/120958	
1:34	*TMEM67*	Comp het	NM_153704.5	c.1843T>C;	p.Cys615Arg;	CM094694	Possibly damaging	DC	Deleterious	4.16	29/0/121374	JBTS
				c.628T>C	p.Ser210Pro	NA	Benign	P	Tolerated	4.45	1/0/121256	
	*IQCB1*	Het	NM_001023571.2	c.59delA	NA	NA	NA	NA	NA	4.45	NA	
5:38	*TMEM67*	Comp het	NM_153704.5	c.2498T>C;	p.Ile833Thr	CM090682	Probably damaging	DC	Deleterious	3.49	4/0/121114	JBTS
				c.628T>C	p.Ser210Pro	NA	Benign	P	Tolerated	4.45	1/0/121256	
3:36	*CEP290*	Het	NM_025114.3	c.6320A>G	p.Lys2107Arg	NA	Probably damaging	DC	Tolerated	4.65	2/0/56244	JBTS
	*KIF7*	Het	NM_198525.2	c.505C>T	p.Arg169Trp		Probably damaging	DC	Deleterious	3.42	1/0/21750	
2:35	*CEP164*	Het	NM_014956.4	c.151C>T	p.Arg51*	NA	NA	DC	NA	2.37	2/0/106462	JBTS
29:65	*NINL*	Het	NM_025176.5	c.1318G>C	p.Glu440Gln	NA	Probably damaging	DC	Damaging	4.6	NA	NPH
4:37	NA											JBTS
10:44	NA											NPH

No candidate variants were identified for two patients (4:37 and 10:44) with Joubert syndrome and isolated nephronophthisis, respectively

*Hom* homozygous; *Het* heterozygous; *Comp het* compound heterozygous; *NA* not available; *DC* disease causing; *P* polymorphism; *NPH* isolated nephronophthisis; *BBS* Bardet-Biedl syndrome; *JBTS* Joubert syndrome

^a^ExAC: allele count/number of homozygotes/allele number [24]

^b^Predicted attenuation canonical splice acceptor site (Human Splicing Finder version 3.0) [25]

Additional biallelic variants were detected in *TMEM67* in one patient with Bardet-Biedl-like syndrome (23:58). Two unrelated patients with Joubert syndrome (1:34 and 5:38) harbored a compound heterozygous known pathogenic variant and the same missense variant of unknown significance (c.[628T>C] p.[Ser210Pro]) in *TMEM67*, suggesting a possible founder effect. This variant represents a novel candidate variant for NPH-RC. Interestingly, the latter two patients had progressive liver fibrosis, liver function abnormalities, and hepatomegaly, which is consistent with reports of liver involvement in patients with mutations in *TMEM67* [6, 14].

### Gene-phenotype associations

#### NPHP1 (*n* = 16)

Biallelic mutations in *NPHP1* were detected in patients with isolated NPH (*n* = 12), Joubert syndrome (*n* = 2), and Senior-Løken syndrome (*n* = 2)*.* Median reported age of symptom onset was 9 years (range 6–26 years) and median age of ESRD was 14 years (range 7–28 years). Patients with *NPHP1* mutations had higher reported frequencies of polyuria (9/10; 90%), polydipsia (8/10; 80%), and enuresis (3/8; 38%) compared to all patients. Strikingly, renal cysts were present in only 4/12 patients (33%) with *NPHP1* mutations, while hyperechogenicity (7/10; 70%) and abnormal corticomedullary differentiation (3/8; 38%) were reported more frequently compared to the cohort average. Extrarenal manifestations included visual impairment (3/16; 19%)—mainly retinitis pigmentosa (*n* = 2)—obesity (*n* = 2), congenital heart disease (*n* = 1), oculomotor apraxia (*n* = 1), and developmental delay (*n* = 1).

#### NPHP4 (*n* = 3)

Three patients with mutations in *NPHP4* presented with isolated NPH. Median reported age of symptom onset was 16 years (range 8–24 years). One patient already had ESRD when the diagnosis was made at age 8 years. The other two patients had not yet developed ESRD at age 19 and 41 years respectively. The renal phenotype was characterized by polydipsia and/or polyuria (*n* = 2) and increased echogenicity (*n* = 2), cysts (*n* = 1), and abnormal corticomedullary differentiation (*n* = 1) on renal ultrasound. There were no extrarenal features reported in patients with mutations in *NPHP4*.

#### WDR35 (*n* = 3)

Three patients had biallelic mutations in *WDR35*; two patients (siblings) had cranioectodermal dysplasia (CED) and one patient had a ciliopathy phenotype that did not constitute a recognizable syndrome. The patients with CED did not yet develop ESRD at age 17 (CKD stage 3) and 12 years (CKD stage 1–2) respectively. Their phenotype was characterized by a narrow thorax and short limbs. The patient without a syndrome diagnosis developed ESRD at age 12 years. Extrarenal manifestations comprised retinitis pigmentosa, oculomotor abnormalities, autism spectrum disorder, liver cirrhosis, polydactyly, and obesity.

#### BBS1 (*n* = 2)

Biallelic mutations in *BBS1* were identified in two patients (siblings) with Bardet-Biedl syndrome and slowly progressive kidney disease. The patients had not yet developed ESRD at age 20 (CKD stage 1–2) and 16 years (CKD stage 2) respectively. Kidney cysts were present in the eldest patient. Both had visual impairment, developmental delay, and obesity.

#### Other genes

Biallelic mutations in the ciliopathy-associated genes *AHI1*, *BBS10*, *IQCB1*, and *OFD1* were identified in single patients. Renal and extrarenal findings associated with mutations in these genes are summarized in Tables 1 and 2.

## Discussion

We describe a cohort of 40 Dutch patients with NPH-RC included in the Nephronophthisis Registry over a period of 3 years (2014 to 2017). This study represents a comprehensive characterization of renal and extrarenal ciliopathy-associated features and associations with genetic findings in a well-defined population. Increased echogenicity was a more prevalent finding on renal ultrasound than kidney cysts (65 vs. 43%), especially in patients with mutations in *NPHP1* (70 vs. 33%). This finding is consistent with literature that describes increased echogenicity and a loss of corticomedullary differentiation on renal ultrasound in 78% of pediatric patients, and cystic lesions in only 51% [6]. Increased echogenicity is considered a sensitive marker for early stages of CKD in children, although it is not specific for NPH [11]. With the Nephronophthisis Registry, we aim to contribute to the growing understanding of NPH-RC phenotypes [6, 7, 13, 14], which will in turn advance diagnostics and genetic counseling.

The median age of NPH symptom onset in the cohort presented here was 9 years (range 5–33 years), which is above the age of 6 years reported in the literature [3]. In addition, while the median age at which ESRD was reached (13 years) was consistent with literature [3, 7–9], the age of ESRD onset varied from 5 to 47 years. Four patients with mutations in *NPHP1* developed ESRD at or after age 20 years. A recent report of *NPHP1*-associated adult onset NPH also supports a later onset of NPH than was previously assumed [26]. This is important because monitoring of renal function in syndromic ciliopathy patients is often discontinued if no kidney disease is present at age 18 years. Based on the range of reported age of onset in our cohort, presymptomatic monitoring of renal function should be performed annually in ciliopathy patients until at least age 30 years. If CKD is established, the monitoring frequency can be adjusted according to the rate of kidney function decline.

Individual patient reports demonstrated diagnostic delay in patients with isolated NPH. For example, a patient with Senior-Løken syndrome caused by *NPHP1* mutations (11:45) had secondary enuresis nocturna for at least 1 year prior to her diagnosis at age 13. Around the time of diagnosis, a renal biopsy showed chronic tubulointerstitial nephritis and glomerulosclerosis. While typical histologic findings, including tubulointerstitial fibrosis, thickened and disrupted tubular basement membrane, and sporadic corticomedullary cysts have been described in NPH [7, 27, 28], interstitial fibrosis, secondary glomerulosclerosis, and tubular atrophy are hallmarks of ESRD of any etiology. Because NPH is typically first suspected in advanced stages of CKD when damage is extensive, histology is often inconclusive and of limited diagnostic value, while it is an invasive procedure for the patient that carries an even higher risk for complications in advanced stages of chronic kidney disease [29]. Another patient with NPH (29:65) presented with polydipsia and polyuria at age 7, when the estimated glomerular filtration rate (eGFR) was normal. At age 15, she presented with fatigue for several weeks and was diagnosed with renal failure. These examples illustrate that a potential treatment window exists if initial signs of NPH are recognized. In addition, early genetic testing and identification of biomarkers that are more sensitive than eGFR could benefit timely detection and adequate supportive treatment, including treatment of anemia and hypertension [20], in NPH-RC patients.

Persistent fatigue and muscle complaints were identified as new presenting symptoms of NPH that should prompt evaluation of kidney function. Muscle complaints could represent musculoskeletal pains, a uremic symptom which is highly prevalent in patients with CKD [30], or could result from electrolyte disturbances (for example low serum magnesium). Electrolyte data was not available for the respective patients at the time of symptoms onset. Hypertension was an initial sign in 21% of patients (*n* = 5), including one patient who currently has CKD stage 2. This finding contradicts literature that suggests that hypertension is a late finding in NPH because of renal salt wasting [31]. However, because of the diagnostic delay in NPH, hypertension could be an initial sign indicating advanced CKD. Diagnostics of NPH can be challenging, even when a causal mutation in a ciliopathy-associated gene has been detected, because other kidney diseases can mimic the NPH phenotype [11]. We detected homozygous mutations in the ciliary gene *TTC21B* in four patients from one family with a renal ciliopathy phenotype comprising renal insufficiency and retinitis pigmentosa (Supplementary Results 1). However, early proteinuria indicated glomerular kidney disease, which has also been described in patients with *TTC21B* mutations [32, 33], instead of NPH. Consequently, knowledge of the NPH phenotype is critical, not only for early detection but also for proper distinction of NPH from phenocopies.

Eight out of 13 NPH-RC patients that were genetically tested in this study remained unsolved, consistent with the less than 50% mutation detection rate described in literature [11, 12]. We detected single heterozygous variants in ciliopathy-associated genes in two patients (15%). A patient with Joubert syndrome (3:36) carried heterozygous variants in *CEP290* and *KIF7*. Another patient with Joubert syndrome (2:35) carried a heterozygous predicted deleterious variant in *CEP164*. Sequencing and deletion/duplication analysis using YouMAQ did not reveal additional variants on the second allele. Single heterozygous mutations in ciliopathy-associated genes have been frequently reported in literature [12, 34]. Possible explanations are that variants on the second allele were missed by the respective sequencing techniques, for example because of intronic localization, that detected heterozygous variants represent genetic modifiers, or that detected variants are unrelated to the phenotype. We also identified a heterozygous predicted deleterious variant in the previously described candidate ciliary gene *NINL* in a patient with isolated NPH (29:65). NINL, or ninein-like protein, colocalizes with CC2D2A at the base of the cilium. A heterozygous variant in *NINL* has been proposed to act as a genetic modifier in a patient with a severe form of Joubert syndrome caused by mutations in *CC2D2A* [35]. Further research is required to elucidate the role of *NINL* in NPH-RC.

We did not find statistically significant genotype-phenotype correlations as a result of the size and heterogeneity of our cohort. However, patterns can be discerned that support previously published gene-phenotype associations. Patients with biallelic *NPHP1* and *NPHP4* mutations exhibited isolated NPH more frequently (in 75 and 100%, respectively) compared to NPH patients with other gene defects, which is in line with previous literature [6, 7, 14]. Visual impairment was the most common extrarenal manifestation in patients with *NPHP1* mutations, present in 19%. Neurologic symptoms (oculomotor apraxia and developmental delay) were reported less frequently than in literature (*n* = 1; 7%) [6, 7, 14]. Interestingly, we found an older median age of symptom onset of 16 years (range 8–24 years) and a lower rate of CKD progression in patients with *NPHP4* mutations compared to patients with other gene defects, corroborating the findings of König et al. [6]. Larger, well-phenotyped cohorts of NPH-RC patients are required to strengthen genotype-phenotype correlations, which is essential for providing an accurate prognosis to patients. To this end, the Nephronophthisis Registry described here can be combined with other international cohorts.

In conclusion, this study provides a comprehensive analysis of renal and extrarenal ciliopathy phenotypes in a cohort of 40 Dutch patients with NPH-RC. Persistent fatigue, polyuria, hypertension, and muscle cramps were identified as initial signs and symptoms of NPH that should prompt timely evaluation of renal function. Symptom onset can occur in adulthood, which supports continuation of presymptomatic kidney function monitoring in patients with a syndromic ciliopathy until at least age 30 years. Our findings confirm that increased echogenicity is a more prevalent finding than cysts on renal ultrasound in NPH-RC patients and that mutations in *NPHP4* are associated with slower kidney disease progression and a lower prevalence of extrarenal manifestations than mutations in other NPH-associated genes. Ultimately, improving recognition of NPH and associated phenotypes will enable adequate surveillance and clinical care for patients with NPH-RC.

## Electronic supplementary material

ESM 1 (DOCX 59 kb)

ESM 2 (DOCX 116 kb)

ESM 3 (DOCX 85 kb)
