# Supplementary material for: Clinical and genetic analyses of a Dutch cohort of 40 patients with a nephronophthisis-related ciliopathy
Source: Pediatr Nephrol. 2018 Jul 5;33(10):1701–12. doi: 10.1007/s00467-018-3958-7 (PMC6132874; doi:10.1007/s00467-018-3958-7)
Supplement: Supplementary file 1 — (DOCX 59 kb) [file 467_2018_3958_MOESM1_ESM.docx]

**Supplementary Methods S1. Deletion/duplication analysis**

For nine patients without a molecular diagnosis at the time of inclusion (1:34, 3:36, 5:38, 6:39, 9:42, 9:43, 11:45, 24:60 and 29:65), deletion/duplication analysis of *NPHP1* was performed using multiplex ligation-dependent probe amplification (MLPA). MLPA analysis was performed in our EN ISO 15189:2012 accredited DNA diagnostics laboratory. The test was performed according to the manufacturer’s protocol (MRC-Holland b.v.) and data were analyzed using the Genemarker program (software version 2.6.0, SoftGenetics LLC).

For one patient (10:44), deletion/duplication analysis was performed using the Flagged youMAQ kit (Multiplicom, Agilent Technologies, Niel, Belgium) according to the manufacturer’s instructions. Data were analyzed using the Genemarker program (software version 2.6.0, SoftGenetics LLC). In addition, the Flagged youMAQ kit was used to assess copy number variations in ciliary genes in which we identified single heterozygous mutations.

**Supplementary Methods S2. Targeted next-generation sequencing**

**Gene panel sequencing**

NGS-based NPH gene panel analysis was performed in one patient (10:44) in our EN ISO 15189:2012 accredited DNA diagnostics laboratory. In brief: A custom SureSelect^XT^ target enrichment assay (Agilent Technologies, elid# 0483791) was used to enrich for the protein coding and flanking intronic sequence of 15 genes known to be associated with NPH: *CEP164*, *CEP290*, *GLIS2*, *INVS*, *IQCB1*, *NEK8*, *NPHP1*, *NPHP3*, *NPHP4*, *RPGRIP1L*, *SDCCAG8*, *TMEM67*, *TTC21B*, *WDR19*, *ZNF423*. Sequencing was performed on a SOLiD^TM^ 5500XL sequencer (50 basepair single read, Life Technologies/Applied Biosystems), with a minimal median coverage of 100X for the targeted bases. To reach a genotyping accuracy of >99%, a minimal vertical coverage of 15 unique reads per base was requested. To deliver a comprehensive analysis of the genes collected in the gene panel, an average horizontal coverage of at least 95% of targeted bases in the gene panel was requested. The horizontal coverage for our patient was 98.3%.

**Whole-exome sequencing**

Whole-exome sequencing was performed in 11 patients. Fragment library preparation and exome target enrichment using the SureSelect 50 Mb (V4) All Exon Kit (Agilent Technologies, CA, USA) was performed as described previously [Harakalova et al., 2011]. Sequencing of the first seven barcoded samples (1:34, 2:35, 3:36, 4:37, 5:38, 9:42 and 9:43) was performed on the SOLiD 5500XL platform (Life technologies, Carlsbad, CA, USA). Average coverage of the exome was 75X. Reads were processed and aligned to the human reference genome (hg 19). Variant calling and annotation were performed using our custom analysis pipeline as described previously [Nijman et al., 2010]. Variants were filtered based on coverage (>8 reads), percentage of the variant in exome reads (>70 for homozygous variants and >15 for heterozygous variants) and population allele frequency below 1% in publicly available genomic databases including Exome Variant Server (ref), USCS Genome Browser (Kent et al Genome Res 2002) and the ExAC Browser (Lek et al Nature 2016), and prioritized based on predicted effect using Polyphen2 (Adzhubei et al Nat Methods 2010), SIFT (Kumar et al Nat Protoc 2009) and MutationTaster2 (Schwarz et al Nat Methods 2014), and relation to cilia based on our in-house database, CiliaCarta (Van Dam BioRxiv 2017) and a Pubmed search.

For the latter four patient samples (10:44, 11:45, 23:58 and 29:65), exomes were enriched using the SureSelect XT Human All Exon V5 kit (Agilent) and sequenced in rapid run mode on the HiSeq2500 sequencing system (Illumina) at a mean target depth of 100X. The target was defined as all coding exons of UCSC and Ensembl +/- 20bp intron flanks. At this depth ~95% of the target was covered at least 15X. Reads were aligned to hg19 using BWA (BWA-MEM v0.7.5a) and variants were called using the GATK haplotype caller (v2.7-2). Detected variants were annotated, filtered and prioritized using the Bench NGS Lab platform (Cartagenia, Leuven, Belgium). For patient 23:58, trio analysis of patient and both parents was performed. Analysis was based upon a tiered analysis approach. The first tier analyzed known intellectual disability genes. The second tier filtered for *de novo* variants and the last tier filtered for recessive variants. Variant confirmation and segregation analyses were performed using standard Sanger sequencing (primer sequences available upon request).
