# Supplementary material for: Clinical and genetic analyses of a Dutch cohort of 40 patients with a nephronophthisis-related ciliopathy
Source: Pediatr Nephrol. 2018 Jul 5;33(10):1701–12. doi: 10.1007/s00467-018-3958-7 (PMC6132874; doi:10.1007/s00467-018-3958-7)
Supplement: Supplementary file 2 — (DOCX 116 kb) [file 467_2018_3958_MOESM2_ESM.docx]

**Supplementary Results S1. Twelve patients with a renal ciliopathy phenotype**

Twelve out of 52 patients had a renal ciliopathy without sufficient evidence for NPH. Of these, six patients had a perinatally lethal phenotype, including four patients from one family with bilateral renal agenesis or renal hypoplasia due to homozygous mutations in *KIF14* [1], and two patients from two families with severe cystic kidney disease and anhydramnios sequence due to compound heterozygous mutations in *BBS10* and *BBS1* respectively (**Supplementary Table S2**). Severe Meckel-Gruber syndrome-like phenotypes caused by mutations in Bardet-Biedl syndrome-associated genes, including *BBS1*, have been described previously [2, 3]. Interestingly, a sibling of the patient with *BBS1* mutations, who had the same *BBS1* genotype, had mild developmental delay, polydactyly and no renal disease at age 3 years, demonstrating vast intrafamilial variability of phenotypes. Two other patients had a ciliopathy phenotype that included kidney cysts or unilateral kidney agenesis. Finally, four patients from one family had retinitis pigmentosa and renal insufficiency in combination with proteinuria due to mutations in *TTC21B*. Although mutations in *TTC21B* are known to cause nephronophthisis type 12, biallelic mutations in *TTC21B* have also been identified in patients with glomerulopathies [4, 5]. Because of the presence of proteinuria early in the disease course, we could not exclude glomerular kidney disease and our patients did not meet diagnostic criteria of nephronophthisis. These twelve patients highlight the spectrum of renal ciliopathy phenotypes and demonstrate that the distinction between NPH (especially infantile NPH, which can present with enlarged, cystic kidneys antenally) and other renal ciliopathy phenotypes can pose challenges [6][7].

**References:**

1. Filges I, Nosova E, Bruder E, et al (2014) Exome sequencing identifies mutations in KIF14 as a novel cause of an autosomal recessive lethal fetal ciliopathy phenotype. Clin Genet 86:220–228. doi: 10.1111/cge.12301

2. Ashkinadze E, Rosen T, Brooks S, et al (2013) Combining fetal sonography with genetic and allele pathogenicity studies to secure a neonatal diagnosis of Bardet– Biedl syndrome. Clin Genet 83:553–559. doi: 10.1002/nbm.3066.Non-invasive

3. Karmous-Benailly H, Martinovic J, Gubler M, et al (2005) Antenatal presentation of Bardet-Biedl syndrome may mimic Meckel syndrome. Am J Hum Genet 76:493–504. doi: 10.1086/428679

4. Bullich G, Vargas I, Trujillano D, et al (2016) Contribution of the TTC21B gene to glomerular and cystic kidney diseases. Nephrol Dial Transpl 0:1–6. doi: 10.1093/ndt/gfv453

5. Stokman MF, Renkema KY, Giles RH, et al (2016) The expanding phenotypic spectra of kidney diseases: Insights from genetic studies. Nat Rev Nephrol 12:472–483. doi: 10.1038/nrneph.2016.87

6. Salomon R, Saunier S, Niaudet P (2009) Nephronophthisis. Pediatr Nephrol 24:2333–44. doi: 10.1007/s00467-008-0840-z

7. Oud MM, van Bon BW, Bongers EMHF, et al (2014) Early presentation of cystic kidneys in a family with a homozygous INVS mutation. Am J Med Genet Part A 164:1627–1634. doi: 10.1002/ajmg.a.36501
