# Supplementary material for: Clinical and genetic analyses of a Dutch cohort of 40 patients with a nephronophthisis-related ciliopathy
Source: Pediatr Nephrol. 2018 Jul 5;33(10):1701–12. doi: 10.1007/s00467-018-3958-7 (PMC6132874; doi:10.1007/s00467-018-3958-7)
Supplement: Supplementary file 3 — (DOCX 85 kb) [file 467_2018_3958_MOESM3_ESM.docx]

**Supplementary Table S1. Clinical findings in cohort of 40 patients with nephronophthisis-related ciliopathies**

| ID | M/F | Age | Gene | Zygosity | Renal phenotype | Visual impairment^1^ | Oculomotor abnormalities | Developmental delay | Liver | Congenital heart defect | Narrow thorax | Poly-dactyly | Obesity | Additional information |
| --- | --- | --- | --- | --- | --- | --- | --- | --- | --- | --- | --- | --- | --- | --- |
| 39:79 | M | 25 | *AHI1* | Comp het | NPH | + | + | + | - | - | - | - | - | Scoliosis |
| 54:98 | M | 20 | *BBS1* | Hom | NPH | + | NA | + | NA | NA | - | - | + | Benign hydrocephalus |
| 54:99 | F | 16 | *BBS1* | Hom | NPH | + | - | + | NA | NA | - | - | + |  |
| 18:52 | M | 13 | *BBS10* | Hom | NPH | + | NA | + | - | - | - | + | + | Genital abnormality |
| 48:91 | M | 29 | *IQCB1* | Hom | NPH | + | + | - | NA | NA | - | - | - | Multinodular goiter |
| 6:39 | M | 62 | *NPHP1* | Hom | NPH | - | - | - | - | - | - | - | - | Hearing impairment |
| 9:42 | M | 18 | *NPHP1* | Comp het | NPH | - | - | - | NA | NA | NA | NA | + |  |
| 9:43 | F | 24 | *NPHP1* | Comp het | NPH | - | - | - | - | - | - | - | - |  |
| 11:45 | F | 35^2^ | *NPHP1* | Comp het | NPH | + | - | - | - | - | - | - | + |  |
| 12:46 | F | 34 | *NPHP1* | Hom | NPH | - | - | - | - | - | - | - | - |  |
| 14:48 | M | 37 | *NPHP1* | Hom | NPH | +^3^ | - | - | - | - | - | - | - |  |
| 15:49 | F | 16 | *NPHP1* | Comp het | NPH | - | - | - | NA | NA | - | - | - |  |
| 16:50 | F | 9 | *NPHP1* | Hom | NPH | - | - | - | - | +^4^ | - | - | - |  |
| 22:57 | F | 31 | *NPHP1* | Hom | NPH | + | - | - | NA | - | - | - | - | Diaphragmatic hernia |
| 24:60 | M | 32 | *NPHP1* | Hom | NPH | - | - | - | - | - | - | - | - |  |
| 32:71 | F | 33 | *NPHP1* | Comp het | NPH | - | NA | NA | - | - | NA | NA | - |  |
| 34:73 | M | 13 | *NPHP1* | Hom | NPH | - | + | + | NA | - | - | - | - | Hypodontia |
| 34:74 | M | 10 | *NPHP1* | Hom | NPH | - | NA | NA | NA | NA | - | - | - |  |
| 38:78 | F | 12 | *NPHP1* | Comp het | NPH | - | - | - | NA | - | - | - | - |  |
| 49:92 | F | 18 | *NPHP1* | Hom | NPH | - | - | - | NA | - | - | - | - |  |
| 55:00 | F | 9 | *NPHP1* | Hom | NPH | - | - | - | NA | - | - | - | - |  |
| 19:53 | M | 11 | *NPHP4* | Hom | NPH | - | - | - | - | - | NA | - | - |  |
| 35:75 | F | 41 | *NPHP4* | Comp het | NPH | - | - | - | NA | NA | NA | NA | - | Astma |
| 53:97 | F | 19 | *NPHP4^5^* | Comp het | NPH | - | - | - | NA | - | - | - | - |  |
| 41:81 | M | 12 | *OFD1* | Hem | NPH | - | + | + | NA | - | - | - | - |  |
| 46:88 | M | 17 | *WDR35* | Comp het | NPH | - | - | - | NA | - | + | - | - | Short limbs |
| 46:89 | F | 12 | *WDR35* | Comp het | NPH | - | - | - | - | - | + | - | - |  |
| 60:06 | F | 33 | *WDR35* | Comp het | NPH | + | + | - | + | - | - | + | + | Pancreas cysts, ovarium cysts, neonatal teeth, bronchiectasis |
| 1:34 | M | 17 | -^6^ | - | NPH | + | + | + | + | NA | NA | NA | - | Abnormal breathing pattern |
| 2:35 | F | 16 | -^6^ | - | NPH | + | + | + | - | NA | - | + | + | Coloboma, tongue and mandible polyps, short limbs |
| 3:36 | M | 24 | -^6^ | - | NPH | - | + | + | NA | NA | NA | + | - | Scoliosis |
| 4:37 | F | 17 | -^6^ | - | NPH | + | + | + | + | NA | - | - | NA | Abnormal breathing pattern, coloboma |
| 5:38 | M | 41 | -^6^ | - | NPH | + | + | + | + | NA | NA | NA | NA | Abnormal breathing pattern, coloboma |
| 10:44 | M | 17 | -^6^ | - | NPH | - | - | - | - | NA | - | - | - |  |
| 23:58 | F | 15 | *-*^6^ | - | NPH | - | - | + | - | - | - | - | + |  |
| 27:63 | F | 19 | -^7^ | - | NPH | + | NA | + | + | + | NA | NA | + |  |
| 29:65 | F | 24 | -^6^ | - | NPH | - | - | - | - | - | - | - | - |  |
| 33:72 | M | 51 | -^7^ | - | NPH | + | NA | NA | NA | NA | NA | NA | - |  |
| 44:86 | F | 23 | -^7^ | - | NPH | - | - | - | - | - | - | - | - |  |
| 51:94 | M | 54 | -^7^ | - | NPH | + | NA | NA | NA | - | NA | NA | NA | Bilateral inguinal hernia |

M: male, F: female, +: present, -: absent, NA: not available, Hom: homozygous, Comp het: compound heterozygous, Hem: hemizygous.
^1^Visual symptoms include night blindness, retinitis pigmentosa, constricted visual fields and ocular coloboma.
^2^Died from non-nephronophthisis-related cause.
^3^Abnormal electroretinography and constricted visual fields with normal findings on fundoscopy.
^4^Left-sided superior vena cava.
^5^Compound heterozygous known pathogenic variant and a variant of unknown significance in *NPHP4.*^6^Findings from genetic testing described in Table 3.
^7^Genetic diagnosis not available and no consent for genetic testing within this study.

**Supplementary Table S2. Clinical findings in twelve patients with a renal ciliopathy**

| ID | M/F | Age | Gene | Zygosity | Renal phenotype | Visual impairment^1^ | Oculomotor abnormalities | Developmental delay | Liver | Congenital heart defect | Narrow thorax | Poly-dactyly | Obesity | Additional information |
| --- | --- | --- | --- | --- | --- | --- | --- | --- | --- | --- | --- | --- | --- | --- |
| 36:76 | M | 40 | *BBS1* | Hom | Cysts | + | NA | + | NA | NA | - | + | + |  |
| 30:66 | F | 4 | - | - | Unilateral agenesis | + | + | + | - | - | - | + | - | Hearing impairment |
| 42:82 | M | 41 | *TTC21B* | Hom | Renal insufficiency, proteinuria | + | NA | NA | NA | - | NA | NA | NA | Astma |
| 42:83 | F | 33 | *TTC21B* | Hom | Renal insufficiency, proteinuria | NA | NA | NA | NA | - | NA | NA | + |  |
| 42:84 | F | 43 | *TTC21B* | Hom | Renal insufficiency, proteinuria | + | NA | NA | NA | + | NA | NA | NA |  |
| 42:96 | F | 35 | *TTC21B* | Hom | Renal insufficiency, proteinuria | NA | NA | NA | NA | NA | NA | NA | NA | Astma |
| 57:03 | F | NA^1^ | *BBS1* | Comp het | Polycystic kidneys | NA | NA | NA | NA | NA | NA | NA | NA |  |
| 59:05 | M | NA^1^ | *BBS10* | Comp het | Polycystic kidneys, dysplasia | NA | NA | NA | - | - | - | + | NA | Anhydramnios sequence |
| 31:67 | M | NA^1^ | *KIF14* | Hom | Bilateral agenesis | NA | NA | NA | - | - | - | - | - | Anhydramnios sequence |
| 31:68 | F | NA^1^ | *KIF14* | Hom | Cystic hypodysplasia | NA | NA | NA | - | - | - | - | - | Anhydramnios sequence |
| 31:69 | M | NA^1^ | *KIF14* | Hom | Cystic hypodysplasia | NA | NA | NA | - | + | - | - | - | Anhydramnios sequence |
| 31:70 | M | NA^1^ | *KIF14* | Hom | Bilateral hypoplasia | NA | NA | NA | - | - | + | - | - | Anhydramnios sequence |

M: male, F: female, +: present, -: absent, NA: not available, Hom: homozygous, Comp het: compound heterozygous. ^1^Termination of pregnancy.

**Supplementary Table S3. Renal phenotypes in 40 patients with NPH-RC**

| ID Fam: case | Sex | Age (yrs) | Gene | Zygosity | Age symptom onset (yrs) | CKD stage/ Age ESRD (yrs)^1^ | Polyuria | Polydipsia | Enuresis | First symptom | Renal ultrasound findings | | | | |
| --- | --- | --- | --- | --- | --- | --- | --- | --- | --- | --- | --- | --- | --- | --- | --- |
|  |  |  |  |  |  |  |  |  |  |  | Age | Cysts | Abnormal corticomedullary differentiation | Increased  echogenicity | Other structural abnormalities |
| 39:79 | M | 25 | *AHI1* | Comp het | 14 | St. 3 | - | - | - | Weight loss | 23 | - | - | - | - |
| 54:98 | M | 20 | *BBS1* | Hom | NA | St. 1-2 | NA | NA | NA | NA | 17 | + | - | - | - |
| 54:99 | F | 16 | *BBS1* | Hom | NA | St. 2 | - | - | - | NA | 15 | - | - | - | - |
| 18:52 | M | 13 | *BBS10* | Hom | NA | St. 1-2 | + | + | - | NA | 9 | + | + | + | Enlarged kidneys antenatally |
| 48:91 | M | 29 | *IQCB1* | Hom | NA | 10 | NA | NA | NA | NA | NA | NA | NA | NA | - |
| 6:39 | M | 62 | *NPHP1* | Hom | 26 | 28 | + | NA | NA | Fatigue | 27 | + | NA | NA | - |
| 9:42 | M | 18 | *NPHP1* | Comp het | NA | 14 | NA | NA | NA | NA | 14 | - | NA | + | Small kidneys |
| 9:43 | F | 24 | *NPHP1* | Comp het | 9 | 10 | NA | - | - | Fatigue | 10 | - | NA | + | Small kidneys |
| 11:45 | F | 35^2^ | *NPHP1* | Comp het | 12 | 20 | + | + | + | Enuresis | 13 | + | - | + | - |
| 12:46 | F | 34 | *NPHP1* | Hom | 13 | 14 | + | + | - | Polydipsia and polyuria, fatigue | NA | NA | NA | NA | - |
| 14:48 | M | 37 | *NPHP1* | Hom | NA | 26 | NA | NA | NA | Fatigue, muscle cramps | NA | NA | NA | NA | - |
| 15:49 | F | 16 | *NPHP1* | Comp het | 7 | 12 | - | - | + | Hypertension, enuresis | 7 | - | - | + | - |
| 16:50 | F | 9 | *NPHP1* | Hom | 6 | 7 | + | + | NA | NA | 6 | - | NA | + | - |
| 22:57 | F | 31 | *NPHP1* | Hom | NA | 10 | NA | NA | NA | NA | 29 | NA | NA | NA | Nephro-sclerosis |
| 24:60 | M | 32 | *NPHP1* | Hom | NA | 15 | + | + | - | NA | 9 | + | - | - | - |
| 32:71 | F | 33 | *NPHP1* | Comp het | 15 | 22 | NA | NA | - | Fatigue, dizziness | 22 | - | + | + | - |
| 34:73 | M | 13 | *NPHP1* | Hom | 6 | St. 3 | + | + | + | Growth retardation | 8 | - | - | - | - |
| 34:74 | M | 10 | *NPHP1* | Hom | 9 | NA | + | + | NA | Polydipsia and polyuria | 4 | - | - | - | - |
| 38:78 | F | 12 | *NPHP1* | Comp het | 7 | 7 | + | + | - | Polydipsia and polyuria | 7 | + | + | NA | - |
| 49:92 | F | 18 | *NPHP1* | Hom | 16 | 17 | NA | NA | NA | Anaemia, fatigue | NA | NA | NA | NA | - |
| 55:00 | F | 9 | *NPHP1* | Hom | 8 | 8 | + | + | NA | Fatigue, uremic symptoms | 8 | - | + | + | Small, dysplastic kidneys |
| 19:53 | M | 11 | *NPHP4* | Hom | 8 | 8 | + | + | - | Growth retardation | 8 | NA | - | + | - |
| 35:75 | F | 41 | *NPHP4* | Comp het | 24 | St. 1-2 | - | + | - | Red patches skin | 24 | + | NA | NA | - |
| 53:97 | F | 19 | *NPHP4^3^* | Comp het | 16 | St. 2 | - | - | - | Hypertension, fatigue, muscle ruptures | 17 | - | + | + | - |
| 41:81 | M | 12 | *OFD1* | Hem | 5 | 5 | NA | NA | NA | Anaemia | 5 | - | - | + | - |
| 46:88 | M | 17 | *WDR35* | Comp het | NA | St. 3 | + | + | - | Polydipsia and polyuria | 15 | - | + | + | - |
| 46:89 | F | 12 | *WDR35* | Comp het | NA | St. 1-2 | - | - | - | NA | 10 | + | NA | + | - |
| 60:06 | F | 33 | *WDR35* | Comp het | 9 | 12 | + | NA | NA | Hypertension, polydipsia and polyuria | 11 | + | NA | NA | - |
| 1:34 | M | 17 | -^4^ | - | NA | 9 | NA | + | NA | NA | NA | + | NA | NA | Enlarged kidneys, mild dysplasia |
| 2:35 | F | 16 | -^4^ | - | NA | 8 | - | + | NA | NA | 8 | - | - | - | - |
| 3:36 | M | 24 | -^4^ | - | NA | NA | + | + | NA | NA | NA | + | NA | NA | - |
| 4:37 | F | 17 | -^4^ | - | NA | NA | NA | NA | NA | NA | 12 | - | - | - | - |
| 5:38 | M | 41 | -^4^ | - | NA | 27 | NA | NA | NA | NA | 18 | NA | NA | + | - |
| 10:44 | M | 17 | -^4^ | - | 5 | St. 3 | - | + | + | Polydipsia and polyuria, fatigue | 15 | + | - | + | - |
| 23:58 | F | 15 | *-*^4^ | Comp het | NA | St. 1-2 | - | + | - | Polydipsia and polyuria | 12 | - | - | - | Unilateral renal agenesis |
| 27:63 | F | 19 | -^5^ | - | NA | St. 1-2 | NA | NA | NA | NA | 6 | NA | NA | NA | Small kidneys, unilateral dysplasia |
| 29:65 | F | 24 | -^4^ | - | 7 | 15 | + | + | NA | Polydipsia and polyuria | 15 | + | + | + | Multicystic dysplastic kidneys |
| 33:72 | M | 51 | -^5^ | - | NA | 13 | NA | NA | NA | NA | NA | NA | NA | NA | - |
| 44:86 | F | 23 | -^5^ | - | 19 | St. 4 | - | - | - | Hypertension, fatigue | 20 | - | - | + | - |
| 51:94 | M | 54 | -^5^ | - | 33 | 47 | NA | NA | NA | Hypertension | NA | NA | NA | NA | - |

M: male, F: female, +: present, -: absent, NA: not available, Hom: homozygous, Comp het: compound heterozygous, Hem: hemizygous.
^1^Last reported CKD stage is provided for patients who did not (yet) develop ESRD. Age at which ESRD was first reported is provided for patients with ESRD.
^2^Died from non-nephronophthisis-related cause.
^3^Compound heterozygous known pathogenic variant and a variant of unknown significance in *NPHP4.*^4^Findings from genetic testing described in Table 3.
^5^Genetic diagnosis not available and no consent for genetic testing within this study.

**Supplementary Table S4. Genetic diagnoses in 23 patients**

| ID  Fam: case | Gene | Zygosity | Accession # | c.Position | p.Position | HGMD accession | Clinical diagnosis |
| --- | --- | --- | --- | --- | --- | --- | --- |
| 39:79 | AHI1 | Comp het | NM_017651.4 | c.910_911insA  c.2057_2060delACAA | p.Thr304fs  p.Asn686fs | CI061497  CD080787 | JBTS |
| 54:98 | BBS1 | Hom | NM_024649.4 | c.1169T>G | p.Met390Arg | CM021489 | BBS |
| 54:99 | BBS1 | Hom | NM_024649.4 | c.1169T>G | p.Met390Arg | CM021489 | BBS |
| 18:52 | BBS10 | Hom | NM_024685.3 | c.271dupT | p.Cys91SerfsX5 | CI064666 | BBS |
| 48:91 | IQCB1 | Hom | NM_001023570.3 | c.897_900dupCTTG | p.Ile301fs | CI1211317 | SLS |
| 12:46 | NPHP1 | Hom | NM_000272.2 | c.1-?_2202+?del | p.(?) | CG962922 | NPH |
| 14:48 | NPHP1 | Hom | NM_000272.2 | c.320-?_522+?del (del exon 5),  c.1698-?_1810+?del (del exon 17) | p.(?) | NA | NPH |
| 15:49 | NPHP1 | Comp het | NM_000272.3  NM_001128178.1 | c.1027G>A  c.-29-?_1805+?del | p.Gly343Arg  p.(?) | CM066932  CG962922 | NPH |
| 16:50 | NPHP1 | Hom | NM_000272.3 | c.?_-94_*455_?del | p.(?) | NA | NPH |
| 22:57 | NPHP1 | Hom | NA | deletion | NA | NA | SLS |
| 32:71 | NPHP1 | Comp het | NM_000272.3 | c.1027G>A  c.1-?_2202+?del | p.Gly343Arg  p.(?) | CM066932  CG962922 | NPH |
| 34:73 | NPHP1 | Hom | NM_000272.2 | +/-150 kb deletion | NA | CG1511893 | JBTS |
| 34:74 | NPHP1 | Hom | NM_000272.2 | +/-150 kb deletion | NA | CG1511893 | JBTS |
| 38:78 | NPHP1 | Comp het | NM_000272.2 | c.1027G>A  c.1-?_2202+?del | p.Gly343Arg  p.(?) | CM066932  CG962922 | NPH |
| 49:92 | NPHP1 | Hom | NM_000272.2 | c.?_-94_*455_?del | p.(?) | NA | NPH |
| 55:00 | NPHP1 | Hom | NM_000272.2 | c.1-?_2202+?del | p.(?) | CG962922 | NPH |
| 19:53 | NPHP4 | Hom | NM_015102.4 | c.2932del | p.Glu978fs | NA | NPH |
| 35:75 | NPHP4 | Comp het | NM_015102.4 | c.148delG  c.1892_1895delAGAA | p.Val79fs  p.Gln631fs | CD051764  CD051765 | NPH |
| 53:97 | NPHP4 | Comp het | NM_015102.4 | c.122C>T^1^  c.271T>C | p.Pro41Leu  p.Phe91Leu | NA  CM051569 | NPH |
| 41:81 | OFD1 | Hem | NM_003611.2 | c.872-877delAAGATA | p.Lys291_Asp292del | CD160579 | Other^2^ |
| 46:88 | WDR35 | Comp het | NM_001006657.1 | c.2891delT  c.2623G>A | p.Pro964LeufsX15  p.Ala875Thr | CD105623  CM105621 | CED |
| 46:89 | WDR35 | Comp het | NM_001006657.1 | c.2891delT  c.2623G>A | p.P964LfsX15  p.Ala875Thr | CD105623  CM105621 | CED |
| 60:06 | WDR35 | Comp het | NM_001006657.1 | c.932G>T  c.3396-29_3396-18del^1^ | p.Trp311Leu  p.(?) | CM166931  NA | Other^3^ |

Hom: homozygous, Comp het: compound heterozygous, Hem: hemizygous, NA: not available, NPH: isolated nephronophthisis, JBTS: Joubert syndrome, BBS: Bardet-Biedl syndrome, SLS: Senior-Løken syndrome, CED: cranioectodermal dysplasia.
^1^Variant of unknown significance, predicted to be possibly pathogenic.
^2^Phenotype described as X-linked Joubert syndrome or oral-facial-digital syndrome type I.
^3^Phenotype consisting of nephronophthisis, retinitis pigmentosa, liver cirrhosis and autism spectrum disorder.

**Supplementary Table S5. Reasons for suspected NPH in patients without a genetic diagnosis**

| ID Fam: case | Sex | Age (yrs) |  |
| --- | --- | --- | --- |
| 1:34 | M | 17 | Joubert syndrome, ESRD age 9, signs of a renal concentration defect, cysts on renal ultrasound, exclusion of other causes of renal failure |
| 2:35 | F | 16 | Joubert syndrome, ESRD age 8, signs of a renal concentration defect, exclusion of other causes of renal failure |
| 3:36 | M | 24 | Joubert syndrome, CKD (stage unknown), signs of a renal concentration defect, cysts on renal ultrasound, exclusion of other causes of chronic kidney disease |
| 4:37 | F | 17 | Joubert syndrome, CKD (stage unknown), exclusion of other causes of chronic kidney disease |
| 5:38 | M | 41 | Joubert syndrome, ESRD at age 27, increased echogenicity on renal ultrasound, exclusion of other causes of renal failure |
| 10:44 | M | 17 | CKD stage 3, signs of a renal concentration defect, renal ultrasound showing cysts and increased echogenicity, exclusion of other causes of chronic kidney disease |
| 23.58 | F | 15 | CKD stage 1-2, signs of renal concentration defect, exclusion of other causes of chronic kidney disease. Unilateral renal agenesis does not explain decline in renal function |
| 27:63 | F | 19 | Bardet-Biedl syndrome, CKD stage 1-2, unilateral kidney dysplasia on renal ultrasound, exclusion of other causes of chronic kidney disease |
| 29:65 | F | 24 | ESRD at age 15, signs of a renal concentration defect, renal ultrasound showing cysts, abnormal corticomedullary differentiation and increased echogenicity, exclusion of other causes of renal failure |
| 33:72 | M | 51 | Senior-Løken syndrome, ESRD at age 13 and exclusion of other causes of renal failure |
| 44:86 | F | 23 | CKD stage 4, increased echogenicity on renal ultrasound and exclusion of other causes of chronic kidney disease |
| 51:94 | M | 54 | Bardet-Biedl syndrome, ESRD at age 47 and exclusion of other causes of renal failure |

ESRD: end-stage renal disease, CKD: chronic kidney disease.
